# Health Literacy Concepts, Themes, and Research Trends Globally and in Latin America and the Caribbean: A Bibliometric Review

**DOI:** 10.3390/ijerph20227084

**Published:** 2023-11-20

**Authors:** Alberto Paucar-Caceres, Carlos Vílchez-Román, Silvia Quispe-Prieto

**Affiliations:** 1 Department for Operations, Technology, Events and Hospitality Management, Manchester Metropolitan University, Manchester M15 6BH, UK; 2 Research Department, Centrum Católica Graduate Business School (CCGBS), Pontificia Universidad Católica del Perú (PUCP), Lima 15023, Peru; cvilchez@pucp.edu.pe; 3 School of Nursing, Faculty of Health Sciences, National University Jorge Basadre Grohmann, Tacna 23000, Peru; squispep@unjbg.edu.pe

**Keywords:** bibliometrics, Latin American and Caribbean, health literacy themes, survey

## Abstract

(1) Background: Health literacy (HL) debates have increased significantly in the last two decades. HL concepts/themes and models have achieved substantial development in the US and Europe. Although there have been some efforts to develop HL in Latin America and the Caribbean (LAC), these seem to be few and scattered. This paper reviews and discusses developments of HL concepts and themes globally and in LAC over the last two decades. (2) Purpose: This study aimed to identify the prevalent health literacy concepts/themes deployed globally and in LAC as reported in academic journals from 2005 to 2022. We looked into which fields of knowledge have been informing HL research over the last decades. (3) Methods: We conducted a structured search on the Web of Science (WoS), Scopus, PubMed, and SciELO databases to extract the textual data for bibliometric analysis. We analyzed the textual data with VOSviewer and Biblioshiny to better understand health literacy themes and strands currently being researched in the LAC region. We conducted the searches in two periods: the first in May 2023 and the second in October 2023. (4) Results: The bibliometric study highlighted five WoS categories informing most HL global studies: (i) public environmental occupational health; (ii) environmental sciences; (iii) health policy services; (iv) health care science services; and (v) communication. The two predominant categories in LAC are public environmental occupation health and health policy services. Journals hosting HL publications come from these WoS categories. Themes in HL publications can be organized into four thematic clusters: (i) *analytical* (research designs, analytic techniques, and criteria for examining HL data); (ii) *psychometric* (measurement properties of data collection tools); (iii) *pragmatic* (practical issues related to implementing HL programs); and (iv) *well-being* (effectiveness of HL programs on mental health and illness treatment). (5) Conclusions: There is expanding interest in health literacy among scholars. The number of publications has increased substantially, particularly over the last five years. These are dominated by the Global North. The metrics show that LAC and Africa are trailing in publications. There is an emerging focus on adult literacy, functional/low health literacy, and their effect on improving capabilities, comprehension, and communication regarding health-related topics.

## 1. Introduction

In the last two decades, health literacy (HL) has increased in significance as a defined field [1,2] Initially, it was limited to the biomedical arena, but it is now evolving as multidisciplinary research field comprising medical, psychological, public health, environmental studies, and other aspects of the social sciences. Studies on HL underline its influence on public health strategies and provision. There is also a critical role in reducing health disparities, promoting well-being, and empowering citizens in learning how to prevent noncommunicable diseases and poor mental health [3,4].

It has been widely acknowledged that health literacy has a vital and crucial public education role in facilitating access to and utilization of the full range of public health services [5,6]. Poor health conditions usually indicate low levels of HL [5]. Furthermore, there is a consensus among the public health community and the medical profession that health literacy is pivotal for improving health outcomes for individuals and their communities [7]. Furthermore, more recently, health literacy benefits have been linked with the potential achievements of some of the United Nations SDGs [8]. Health literacy themes, models, strategies, and intervention programs have achieved substantial reach across the US and Europe. In contrast, despite some efforts to develop HL in Latin America and the Caribbean (LAC), these appear to be few and scattered [9,10].

This study aimed to identify the most prevalent HL concepts, themes, and models researched globally and in Latin America and the Caribbean (LAC) reported in academic journals over the last two decades.

Since HL seems to involve various disciplines from a wide spectrum, we started by investigating which knowledge fields or disciplines have been informing HL developments both globally and in LAC. We looked into the concepts and themes that have prevailed in HL developments over the last two decades. We believe that this will help to direct future research, particularly in LAC. The two research questions (RQs) that guided the study were as follows:

RQ1: Which fields of knowledge have informed health literacy’s current developments globally and in LAC?
RQ2: What research themes and concepts in health literacy have evolved globally and in LAC over the last two decades?

The paper is organized as follows: After this introduction, in Section 2, we broadly outline the current concepts related to health, literacy, and HL globally and in LAC. In Section 3, we describe the methods and sources used. The results and their discussion are presented in Section 4. In Section 5, we advance our conclusions. Finally, in Section 6, we reflect on the study’s limitations and present some ideas for further research.

## 2. Health, Literacy, and Health Literacy in Latin America and the Caribbean (LAC)

National population health represents one of the greatest concerns of governments and, understandably, this is seen as fundamental to improving overall well-being. According to the WHO, health can be regarded as “a state of complete physical, mental and social well-being, and not merely the absence of disease” [11]. This definition reveals the changing basic assumptions and that the concept of health has certainly evolved in recent times. As an area of focus, health is no longer viewed as a pure biological, individualist, curative, monocausal concept. It is in a state of becoming a biopsychosocial, preventive, multicausal, and collective concept. This concept incorporates social and personal resources, as well as capacities that make a better life possible [12,13].

### 2.1. Literacy

Generally speaking, literacy underpins the idea of health literacy. According to the United Nations Educational, Scientific and Cultural Organization (UNESCO), literacy is deemed to be a fundamental human right. It is coupled with the right to education, and likewise, it favors human development as an instrument to improve health [12]. Literacy is central for effective and efficient health management. It is directly related to individual and public health as a determining factor [14,15]. This is in addition to recognizing the place of social context and education in the well-being of citizens [16]. After investigating the developments of these concepts, Sørensen, argues that HL is inherently linked to literacy. It implies the knowledge of people, their motivation, and the skills to access, understand, appreciate, and apply relevant health information. This is a means to making judgments and decisions about health care and disease prevention and to promoting a better quality of life [17].

### 2.2. Health Literacy

There has been fervent debate concerning HL over the past two decades. HL studies underline its influence on public health strategies and services, and it has significantly contributed to reducing health disparities and to progressing the overall well-being of citizens. The term “health literacy” was first mentioned by Simonds [13] who argued that health education should be considered a key dimension of social policy [18]. According to Simonds [13], there are three defined responsibilities: the health sector delivering quality health; the contribution from the educational system; and the responsibility of the communication and entertainment industry (with a commitment to promoting public health and supporting healthy lifestyles).

The International Union for Health Promotion and Education (IUHPE) highlights the importance of the practical application of health literacy to guide clinical practice, public health interventions, and public policies for the advancement of global health [14]. A good and adequate provision of health Literacy will allow viable solutions to the challenge of providing high-quality, affordable, and universally accessible care [19,20].

That said, there is no single definition of health literacy [21]. A similar situation occurs when determining the best instrument or tools for assessing health literacy among the general population [22]. There are also exhaustive debates outside this study’s scope. The World Health Organization posits that ‘[Health literacy] is made up of the cognitive and social skills that determine the motivation and ability of individuals to access, understand and use the information to promote and maintain good health’ [16].

### 2.3. Health Literacy in Latin America and the Caribbean (LAC)

We are conscious that the particular environment in LAC will pose specific restrictions in the way the term “health literacy” is understood and practiced. Furthermore, inadequate health literacy represents a significant challenge in many low- and middle-income countries. This is due to the combination of lower overall levels of education and poorly functioning and resource-limited healthcare systems. Therefore, it is vitally important to address the complexity of understanding health interventions to determine if they really meet the needs of the population [19]. To this end, it is fundamental to understand the research progress in the context of each individual country, particularly in a region diverse as Latin America. In their multinational study, McClintock, et. al [23] al recognize the characteristics that each country presents and suggest that cultural diversity should be considered to understand the way people perceive the benefits of HL and that health literacy understanding of a country can be gained from the experiences of its citizens when facing barriers and opportunities for obtaining adequate health literacy [23].

Because this study examined HL in LAC, it is worth clarifying that direct translation into Spanish of the words “literacy” and “literate” poses some problems. First, there is no direct translation into Spanish. Second, the closest literal translation “alfabetización” is associated with learning activities to obtain the most elemental levels of reading and writing. The difficulty in using a direct translation is that both terms, "alfabetización” and "alfabetizado” have strong derogatory social connotations (“analfabeto” is a person that has no schooling and is considered to be an ignoramus). In some Latin American countries, the literal translation of literacy, “literacidad” or “literacia”, has been used. This certainly better describes the implications of health literacy as the process of not only knowing about health but also about taking action upon this understanding and helping to create a better narrative based on these its implications and suggesting interventions. That said, these terms “literacidad” or “literacia” do not sound like proper Spanish terms. In this study and after careful consideration, we have decided to keep the English terminology of “health literacy”, but when we refer to the term in Spanish, we keep the “alfabetización en salud”; however, we are conscious that we do not use it as a derogatory term but as a social process of action and in which language practices (oral, writing, and other modes) are intertwined in their complexity with cultural and social elements in every human group.

Therefore, "health literacy” and “alfabetización en salud” are understood as conveying a relational concept. That is, a concept that involves personal skills and abilities acting under the restrictions, requirements, and complexities of a particular environment in particular situations in which there is a dynamic interaction between individuals, groups of individuals, communities, and organizations [24].

## 3. Materials and Methods

In this study, we conducted a bibliometric review at the country, journal, and publication levels. We designed a search strategy for collecting data; then, we processed the textual data. This was to acquire descriptive statistics and visualizations for the conceptual structure of the analyzed publications. The search and review strategies were not prescriptive. The review loosely followed the typologies of reviews compiled by [25]. It can be seen as a strategy between a scoping review and a systematic review in that sought to “systematically search for, appraise and synthesize research evidence”. Our study provides a “narrative with tabular accompaniment” [25].

### 3.1. Information Sources

We first obtained textual data from two multidisciplinary databases: Scopus and Web of Science (WoS). The list includes two relevant LAC research indexes: SciELO Citation Index and the Emerging Sources Citation Index (ESCI). Although both indexes were not initially included in the WoS core collection, they provided access to a high proportion of LAC research that does not integrate with mainstream science (research papers indexed in Scopus and WoS). We complemented the initial results with searches on the PubMed database to obtain a medical-oriented approach to HL research. We also examined literature/systematic reviews on health literacy. This was to obtain a conceptual framework for comparing conceptual themes introduced in this study.

### 3.2. Data Collection

Inclusion and exclusion criteria: The inclusion criteria were the following: articles published between January 2005 to December 2022. The exclusion criteria were the following: nonarticles (books, thesis, editorials, etc.) and articles published before January 2005 and after December 2022.

Data collection was performed according to the following three steps.

(1) In the first step, a set of commands was used to identify studies on health literacy indexed in Scopus and WoS (filtering out was conducted by document type article) for 2005–2022. We used three HL-related terms retrieved from the controlled vocabulary NLM MeSH [26].

WoS #1: TI = (“consumer health information” OR “health literacy” OR “patient medication knowledge” OR (health W/2 literacy))

Scopus #1: TITLE (“consumer health information” OR “health literacy” OR “patient medication knowledge”).

We constructed one global and LAC research dataset, downloaded each publication’s complete bibliographic information from the first search, and imported it into two Excel files (Scopus = 5773/WoS = 4524).

(2) In the second step, we combined #1 with a search command developed to identify concepts, themes, and topics used in HL research globally and for the LAC region:

WoS #2: TI = (“consumer health information” OR “health literacy” OR “patient medication knowledge” OR (health W/2 literacy)) AND AB = (concept* OR framework OR model* OR theme* OR topic*) AND PY = (2005–2022).

Scopus #2: TITLE (“consumer health information” OR “health literacy” OR “patient medication knowledge”) AND ABS (concept* OR framework OR model* OR theme* OR topic*) AND PUBYEAR > 2004 AND PUBYEAR < 2023.

**Figure 1:**
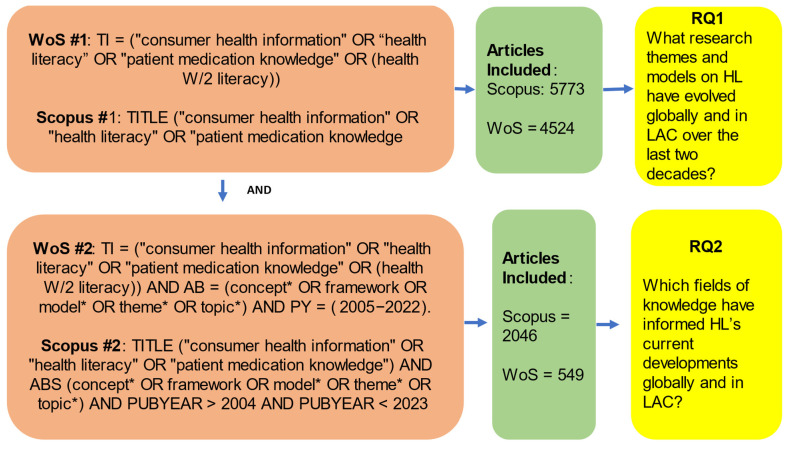
Screening strategy, flowchart of data collection, and research questions.

Then, we exported the results (Scopus = 2046/WoS = 549) as comma-separated and tab-delimited files. The screening strategy, flowchart of data collection, and the linkage to the study research questions (RQs) are presented in Figure 1. We conducted a structured search on the Web of Science (WoS), Scopus, PubMed, and SciELO databases to extract the textual data for bibliometric analysis. We analyzed the textual data with VOSviewer and Biblioshiny to gain a better understanding of the HL themes and strands currently being researched in the LAC region. Searches were conducted in two periods: the first in May 2023 and the second in October 2023.

### 3.3. Data Analysis and Visualization

We obtained frequency and descriptive statistics to answer RQ 1. For RQ 2, we imported text and BibTeX files into VOSviewer 1.6.17 [27] and bibliometrix 4.1.3 [28], two well-known programs for scientific information visualization. Both provide thematic clusters to explore conceptual structures about a topic. The programs apply coword analysis to obtain visual representations [29,30,31], a technique that works with nonmetric multidimensional scaling and cluster analysis. In addition to VOSviewer’s built-in algorithm for filtering out nonsignificant words during the coword analysis, we worked with an ad-hoc list to filter out nonsignificant words such as the name of the months, type of documents, or prefixes. This nonstop list is the thesaurus text file used by VOSviewer. Even though the bibliometrix software is a command-line-based program for R, we used the graphical interface biblioshiny to produce the conceptual mappings. We examined the abstract and keywords with coword analysis and topic modeling to identify the conceptual dimensions of HL literature as reported in LAC publications. For the first analysis, there was a minimum threshold of 10 (WoS) and 30 (Scopus) occurrences of each selected term/keyword. Finally, we compared the visual output of both programs and selected the more relevant map to be included in the results.

By using these results, we tackled the main objectives: (i) to identify the main fields of knowledge that have informed HL development globally and also in the LAC region and; (ii) to identify the concepts, themes, and models used in health literacy approaches in current studies globally and in LAC. Discussion of these results in the context of our research questions are outlined in the next section.

## 4. Results and Discussion

For articles reporting some aspects of health literacy research between 2005 and 2022, the search returned: 14,893 articles from WoS; 6812 from Scopus; 5810 from PubMed; and 207 from SciELO. Table 1 shows a descriptive summary of the search breaking the total into six regions: USA and Canada; the European Union; Asia; Oceania; LAC; and Africa.

### 4.1. Authors Affiliations and Count by Publication Year

As outlined in Table 1, most authors studying health literacy come from countries and regions in the Global North. Notably, the USA/Canada and the EU led the number of publications: WoS returned 1830 and 1559 articles, respectively; Scopus returned 2395 and 2327, respectively; PubMed returned 1481 and 840, respectively; and SciELO returned 15 and 67, respectively. There was only a small group of authors who published in WoS that came from Global South countries in LAC and Africa (144 and 108 respectively). There was a similar situation with Scopus, which had 167 articles from LAC and 153 from articles Africa, as well as with SciELO, which had 138 publications from LAC and six from Africa.

**Figure 2:**
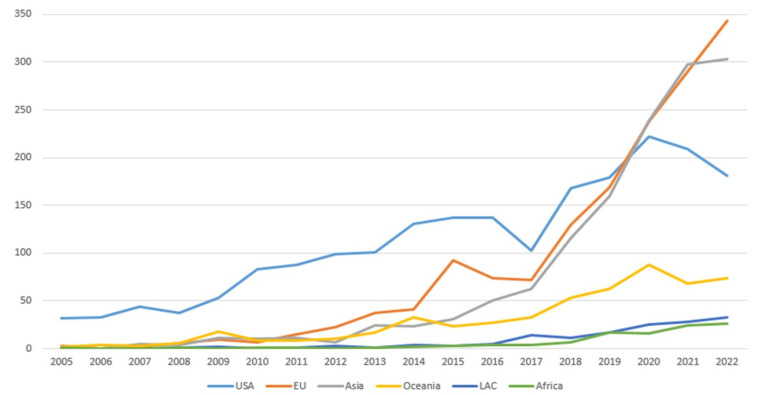
HL Articles published between 2005–2022 per region. SourceL WoS.

Table 2 details the number of publications per region and database searched. Most of the articles are from the USA/Canada and the EU. This disproportion is more noticeable when we observe the historical trends: 5431 articles from the Global North compared to only 258 from the Global South in the WoS database. The situation was the same when Scopus was searched: 5492 articles from the Global North and only 320 from the Global South. In SciELO, LAC and EU consistently produced the highest number of publications, totaling 124 and 59, respectively; Asia, Oceania, and Africa had a smaller presence, with 10, five, and five articles, respectively. Figure 2 shows that for articles indexed by WoS, during the last decades, there has been a steady increase in the overall number of HL publications but more substantially in 2017–2018.

### 4.2. Fields of Knowledge Informing Health Literacy Developments

As stated in the introduction, the first research question of this study aims to ascertain which fields of knowledge have been informing HL development as an area of research and knowledge. To answer this question, we surveyed journals that mention health literacy in the title or abstract fields in the following categories: communication; health policy and services; information science and library science; general and internal medicine; multidisciplinary health care; pediatrics; psychiatry; and public, environmental, and occupational health (SSCI/SCIE). We filtered the count to the top journals occupying the first and second quartiles from the total number of articles in the initial search.

Table 3 contains the list of these twenty-five top journals. The first 13 were indexed in both WoS and Scopus. There were seven indexed only in WoS, and the last five were only found in the Scopus database. We included quartile and associated metrics. We also included the country of the editorial board. The majority of these boards were based in the USA and EU countries. It is also worth noting that 17.2% of the publications (WoS database) were concentrated in the first five journals: *International Journal of Environmental Research and Public Health*; *Journal of Health Communication*, *Patient Education and Counselling*; *BMC Public Health*; and; *PLoS ONE*.

There is some indication of which fields of knowledge have been informing health literacy research by the concentration of HL themes in the journals listed in Table 3. As it can be seen (last column), the scope of the top five journals (WoS indexed) vary in fields of knowledge and include environmental health sciences, health information/literacy, patient education/health care, epidemiology/public health, and multidisciplinary health care. The range of journals indexed by Scopus (only) is somehow a bit more health-related and includes health literacy/public health, health services/digital health, cancer education, health care/diversity/equity, and public health/oral health. This confirms that although HL might have its origins in medical sciences, the social science fields and environmental sciences have clearly influenced and informed its development as a field. Thus, one can argue that HL is becoming a more multidisciplinary arena [32].

### 4.3. Research HL Themes and Concept Developments Globally and in LAC over the Last Two Decades

The second research question driving this study concerns the concepts and themes, and the second research question driving this study concerns the concepts, themes, and models used in HL approaches. Table 4 shows the HL research concepts and themes that have taken shape over the last decades, both globally and in LAC. Our results show that five WoS categories describe most HL global studies during the years 2012 to 2022: (i) public environmental occupational health; (ii) environmental sciences; (iii) health policy services; (iv) health care science services; and (v) communication. In the LAC region, the two predominant categories are public environmental occupation health and health policy services (see lower part of Table 4).

**Figure 3:**
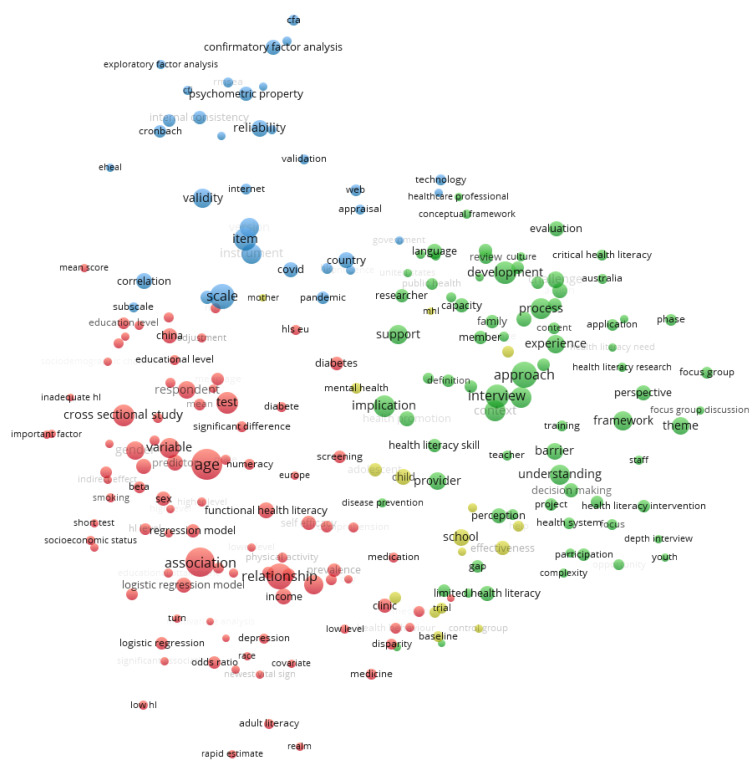
Topics based on coword analysis for global studies. Conceptual structure for words and terms mentioned in the abstract field with a frequency of a minimum of ten occurrences. Source: WoS.

**Figure 4:**
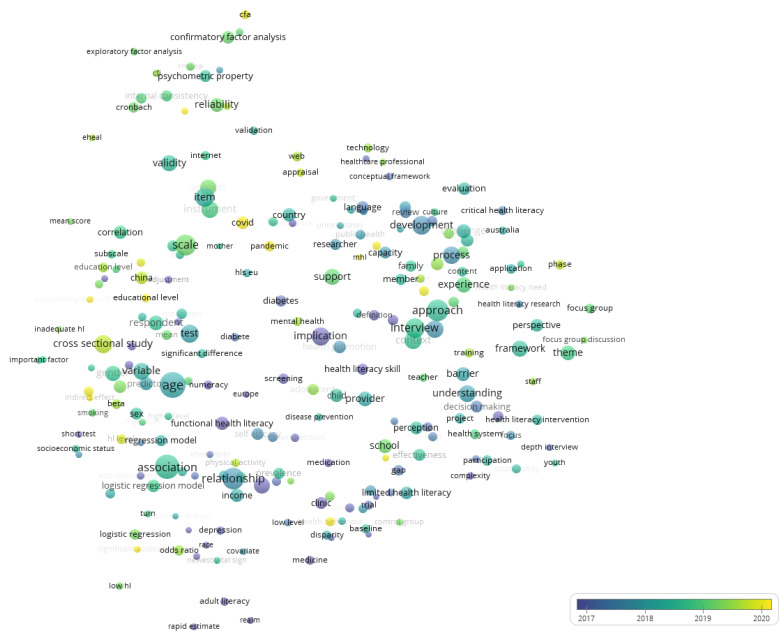
Evolution of topics based on coword analysis for global studies (2017–2020). Conceptual structure for words and terms mentioned in the abstract field with a frequency of a minimum of ten occurrences. Source: WoS.

Figure 3 and Figure 4 highlight the topics based on coword analysis. It maps the structure based on words and terms mentioned in abstracts. Since most journals indexed in Scopus are also indexed in WoS, for space reasons, we have restricted the visualization of concept maps to the search from WoS. Our study provides evidence that HL publications are organized into four thematic clusters. These clusters can be seen in Figure 3: the red cluster for the analytical dimension refers to research designs, analytic techniques, and criteria for examining HL data; the blue cluster for the psychometrical dimension refers to the measurement properties of data collection tools (e.g., validity and reliability) and multivariate analytic techniques (e.g., correlation, confirmatory factor analysis) used to study HL; the green cluster for the pragmatical dimension refers to the practical issues surrounding implementing HL programs (e.g., approach, context, development, framework, and implications of health interventions); finally, the yellow cluster for the well-being dimension refers to the effectiveness of HL programs on mental health and illness treatment.

Figure 4 illustrates how the HL research themes and concepts have evolved over the last three years. In 2017, the topics researched were explicitly related to HL; i.e., “low HL”, “functional HL”, and “adult HL”; these are the variables represented in the dark blue bubbles at the bottom of the figure. More recently, in 2020, the research focus has moved to more contextual areas surrounding HL, such as the “covid” and “pandemic” variables indicated by the yellow bubbles. In between these years (green bubbles in Figure 4), the focus seems to have been on variables such as “age” and “tests” of HL interventions.

In terms of the research hotspots, our results suggest that both globally and in LAC, the following research areas are budding: areas where the *person/patient is in charge or the main operator of HL*; areas of research where *health professionals and care providers* play increasingly important roles; and areas of research where *the role of health services in health literacy* is important. The above echoes the idea that, in future HL research, these three “actors” should be considered: *environments*, *health professionals*, and *people* [33].

It is striking not to find mental health as a central and broad topic of research in health literacy despite the fact that the research has revealed that there are massive gaps in the data surrounding treatment in LAC countries [34]. The WHO is now calling for strengthened information systems, scientific data, and research [35]. This is especially because in the last 3 years, there are reports that the pandemic has led to increased stress, depression, fear, confusion, and the related exacerbation of family violence [36]. Yet, there is a lack of attention (both in public health actions and in research, to integrating HL as a key strategy [37].

## 5. Conclusions

(a) Over the last two decades, scholarly interest in health literacy has grown. Our bibliometric analysis revealed that the number of publications has had a definite surge, especially over the past five years. As may be expected, the number of HL publications is dominated by the Global North, particularly the US/Canada and Europe. It is interesting to note that despite (or maybe because of) the COVID-19 pandemic, the number of publications has remained steady, even showing a slight increase. There is much less vibrancy in the Southern Hemisphere (LAC and Africa).
(b) The bibliometric analysis suggests that HL publications can be organized into four thematic clusters: *analytical* (research designs, analytic techniques, and criteria for examining HL data); *psychometric* (measurement properties of data collection tools); *pragmatic* (practical issues related to implementing HL programs); and *well-being* (effectiveness of HL programs on mental health and illness treatment).
(c) In terms of the concepts, themes, and models used in HL approaches over the last 10 years (2012–2022), our bibliometric study highlights five WoS categories informing most HL global studies: (i) public environmental occupational health; (ii) environmental sciences; (iii) health policy services; (iv) health care science services; and (v) communication. In LAC, the two predominant categories were public environmental occupation health and health policy services. This is consistent with our conclusion that the journals hosting HL publications seem to come from these WoS categories. Although these journals have created a reasonable space for HL-related publications, we argue that, in order for HL to grow as a research topic in its own right, there needs to be more journals fully devoted to HL and a space for journals with a clear HL focus.
(d) HL concepts, themes, and research interest have shifted in focus rapidly over the past years. Initially, HL topics related to the measuring HL were researched; over the years, some HL contextual elements and variables such as age, gender, and the conceptual framework underpinning HL were researched more actively.
(e) The outlets where HL topics are published seem to be dominated by five journals, ranging from *environmental research, public health, communication, and health education*. Because HL is a relatively new area of research and medical practice, HL scholars have used journals such as *International Journal of Environmental Research and Public Health, Journal of Health Communication, International Perspectives, Patient Education and Counselling, BMC Public Health*, and *PLoS ONE*. These journals encourage submissions on topics related to health, environmental sciences, medicine, health communication, and global public health, among others.
(f) Publications of HL research in the above group of journals seem to indicate that HL research is still in its initial stages and despite there being a few health literacy-dedicated journals, the research in this field has been heavily informed by fields belonging to social and natural sciences such as communication sciences and environmental sciences, among others. This seems to signal that future HL research will develop in settings that encourage interdisciplinary research.

## 6. Final Remarks, Contributions, and Research Limitations

The contributions of this study are twofold: First, it identified key health literacy themes and concepts researched over the last two decades both globally and in Latin-America and the Caribbean (LAC). The bibliometric study highlighted five categories informing most HL global studies: (i) public environmental occupational health; (ii) environmental sciences; (iii) health policy services; (iv) health care science services; and (v) communication. The two predominant categories in LAC are (i) public environmental occupation health and (ii) health policy services.

Secondly, the study provided empirical evidence concerning the nature of the developments of HL research by tracing which fields of knowledge have informed its development. We identified the main research outlets involved in HL research: journals whose scope ranges from environmental research, public health, communication, and health education. Our results indicate that there are HL-dedicated journals. Finally, the study draws attention to the multidisciplinary nature of global and LAC health literacy research.

The study has potential limitations that should be noted. Although we tried to complement our searches with the PubMed and SciELO databases, the core of the HL developments discussed here comes mainly from the WoS and Scopus databases. Admittedly, this is biased towards English-language publications. This factor could help to reinforce the idea of a research gap between the Global North and Global South. Given the high volume of Global South studies now being disseminated as open-access publications, open searches might shed light on the development of HL in the Global South. Finally, to better understand the HL landscape and developments, there is a need for an in-depth analysis of the HL-related seminal articles/authors during the last decades.

## Figures and Tables

**Table 1:** Descriptive Summary: HL articles published between 2005 and 2022.

Articles	PubMed	SciELO	Scopus	WoS
Number of Authors	17,415	227	6812	14,893
(1). USA (United States of America/Canada)	1481	15	2395	1830
(2). EU (European Union)	840	67	2327	1559
(3). Asia	1005	10	1177	1352
(4). Oceania (Australia/New Zealand)	415	6	593	539
(5). LAC (Latin America and the Caribbean)	71	138	167	144
(6). Africa	51	6	153	108
(7). Undefined			235	
Total	5810	207	7047	5532

Note: The geographical region recognizes the author’s institutional affiliation.

**Table 2:** Publications of HL per region from 2005 to 2022.

Year	USA				EU				Asia				Oceania				LAC				Africa			
	SciELO	Scopus	WoS	PMed	SciELO	Scopus	WoS	PMed	SciELO	Scopus	WoS	PMed	SciELO	Scopus	WoS	PMed	SciELO	Scopus	WoS	PMed	SciELO	Scopus	WoS	PMed
2005		37	32	33		5	3	1		2	1	1		3	2	1		0	0	0		0	1	0
2006		34	33	70		0	0	1		0	0	1		4	4	15		0	0	0		0	0	0
2007		53	44	132		6	2	2		7	5	4		2	3	16		0	0	0		0	0	0
2008		49	37	185		7	6	2		7	4	7		6	6	21		1	1	0		1	1	1
2009	1	74	53	252	0	16	9	4	0	14	11	10	0	19	18	37	2	2	2	0	0	1	0	1
2010	0	103	83	343	0	11	7	5	0	15	10	11	0	11	8	46	0	1	0	0	0	2	1	2
2011	2	94	88	438	0	14	15	7	0	11	11	14	0	8	8	54	2	2	1	4	0	1	1	3
2012	1	113	99	553	3	23	22	10	0	19	7	15	0	15	10	67	5	5	3	4	0	2	0	4
2013	0	139	101	675	1	42	37	14	0	29	24	37	0	19	17	78	1	0	1	4	0	1	1	5
2014	0	161	131	835	4	55	41	45	0	39	23	67	0	43	33	135	2	4	4	5	0	2	2	6
2015	0	191	137	1169	5	112	92	87	0	54	31	148	0	28	23	223	2	4	3	9	1	4	3	7
2016	1	167	137	1558	5	97	74	145	0	73	50	240	0	28	27	353	4	7	5	23	0	4	4	7
2017	2	173	103	1965	4	139	72	201	0	99	63	336	0	43	33	547	11	25	14	68	1	12	4	14
2018	1	175	168	2443	7	162	130	263	1	132	116	525	1	52	53	854	8	11	11	101	0	4	7	14
2019	2	186	179	2926	2	163	169	353	1	210	160	721	1	55	63	1098	22	19	17	136	1	14	17	18
2020	1	207	222	3468	5	238	238	428	2	265	239	1125	1	69	88	1364	16	23	25	209	1	16	16	24
2021	2	222	209	4003	7	284	290	727	2	359	298	1620	1	81	68	1777	24	27	28	266	0	26	24	39
2022	0	212	181	4758	16	440	343	1040	4	443	303	2309	1	107	74	2263	25	36	33	397	1	36	26	58
Total	13	2390	2037		59	1814	1500		10	1778	1356		5	593	538		124	167	148		5	126	108	

Note: The total is higher than the sum of columns because some papers have institutional affiliations from more than one country. PubMed data was processed with Biblioshiny’s accumulative algorithm. SciELO coverage in WoS began in 2009.

**Table 3:** Fields of knowledge informing HL developments over the last two decades: the top 20 journals with impact factor in Journal Citation Reports (JCR) and Scimago SJR.

Source of Titles	SJR Quartile (2021)	Scopus Articles	JIF Quartile (2021)	WoS Articles (%)	Country Editorial Board	Journal Scope Topics
1. *International Journal of Environmental Research and Public Health*	Q2	268	Q1	267 (5.90)	US	Environmental health sciences
2. *Journal of Health Communication*	Q1	121	Q2	156 (3.45)	US	Health information/literacy
3. *Patient Education and Counseling*	Q2	137	Q2	139 (3.07)	NO	Patient education/health care
4. *BMC Public Health*	Q1	128	Q2	128 (2.83)	US	Epidemiology/public health
5. *PLoS ONE*	Q1	89	Q2	90 (1.99)	US	Multidisciplinary
6. *BMJ Open*	Q1	79	Q2	75 (1.66)	UK	Clinical medicine/epidemiology
7. *Health Promotion International*	Q1	64	Q2	75 (1.66)	AU	Health promotion
8. *Frontiers in Public Health*	Q1	55	Q1	53 (1.17)	UK	Public health/epidemiology
9. *Journal of General Internal Medicine*	Q1	56	Q1	53 (1.17)	US	Primary care/internal medicine
10. *Journal of Medical Internet Research*	Q1	46	Q1	44 (0.97)	CA	Health informatics/health services
11. *Health Expectations*	Q1	22	Q2	25 (0.55)	UK	Health care/health policy
12. *Healthcare*	Q2	22	Q2	22 (0.49)	FI	Health care/medicine
13. *Health Communication*	Q1	24	Q2	21 (0.46)	US	Health communication
14. *Journal of Community Health*			Q1	17 (0.38)	US	Public health/health services
15. *Academic Pediatrics*			Q2	16 (0.35)	US	Pediatrics
16. *BMC Psychiatry*			Q2	16 (0.35)	NL	Psychiatric diseases
17. *European Journal of Public Health*			Q2	16 (0.35)	SE	Public health/multidisciplinary
18. *Health Education & Behavior*			Q2	16 (0.35)	US	Public health/health behavior
19. *Research in Social & Administrative Pharmacy*			Q2	16 (0.35)	US	Health services/medication use
20. *Frontiers in Psychiatry*			Q2	15 (0.33)	DE	Psychiatric diseases
21. *Health Literacy Research and Practice*	Q1	74			US	Health literacy/public health
22. *BMC Health Services Research*	Q1	40			DE	Health services/digital health
23. *Journal of Cancer Education*	Q2	26			US	Cancer education
24. *Health Promotion Practice*	Q2	23			US	Health care/diversity/equity
25. *Journal of Public Health Dentistry*	Q2	21			US	Public health/oral health

Note: SJR—SCImago Journal Rank; JIF—journal impact factor in JCR categories of: communication; health policy and services; information science and library science; general and internal medicine; multidisciplinary care; pediatrics; psychiatry; and public, environmental and occupational health (SSCI/SCIE). WoS’ full list = 4524 publications. US—United States; NO—Norway; UK—United Kingdom; AU—Australia; CA—Canada; DE—Germany; FI—Finland; NL—The Netherlands; SE—Sweden.

**Table 4:** Research concepts and themes on HL have evolved over the last ten years globally and in LAC.

WoS Category in Global Studies	2012	2013	2014	2015		2016		2017		2018		2019		2020		2021		2022		Total	
Public environmental occupational health	7	15	17	18		12		20		21		27		56		72		72		337	
Environmental sciences			1	1				1		8		12		31		25		38		117	
Health policy services	5		6	6		2		10		6		2		7		22		12		78	
Health care sciences services	2		6	5		5				4		4		8		16		11		61	
Communication	7	7	9	6		8		4		3		3		3		3		2		55	
Medicine general internal	2	1	6	4		1		1		4		7		5		7		9		47	
Information and library science	6	7	9	4		7		1		2		3		2		2		2		45	
Social sciences interdisciplinary	1	7	8	1		4		4		4		5		4		6		1		45	
Multidisciplinary sciences	1	1	2	3		3		4		3		4		5		2		4		32	
Medical informatics			1	1		4		1		2		2		4		6		3		24	
Psychiatry		1				1						1		1		3		1		8	
Pharmacology and pharmacy		2	1					1										1		5	
Pediatrics			1							1										2	
Total	31	41	67	49		47		47		58		70		126		164		156			

**WoS Category in LAC Studies**				**2017**			**2018**			**2020**			**2021**			**2022**			**Total**		
Public environmental occupational health				1						1			3			1			6		
Health policy services										1			3			1			5		
Multidisciplinary sciences							1			1									2		
Medicine general internal																1			1		
Total				1			1			3			6			3					

## Data Availability

Full data supporting our results can be found at: https://drive.google.com/drive/u/1/folders/1BUZ9hZEn8iatL_-86ANa1LaLCFi1C-IQ (accessed on 2 November 2023).
